# Rehabilitation after Total Laryngectomy—A Tribute to the Pioneers of Voice Restoration in the Last Two Centuries

**DOI:** 10.3389/fmed.2017.00081

**Published:** 2017-06-26

**Authors:** Kai J. Lorenz

**Affiliations:** ^1^Department of Otolaryngology/Head and Neck Surgery, German Armed Forces Hospital, Ulm, Germany

**Keywords:** total laryngectomy, voice rehabilitation, voice prosthesis, esophageal speech, laryngeal cancer

## Abstract

**Background:**

The most severe consequence of laryngectomy for patients is the loss of their voice. For this reason, voice rehabilitation has been an integral aspect of treatment after total laryngectomy from the very beginning. A wide variety of different technical and surgical approaches are available and reflect the problems associated with the rehabilitation of communication and swallowing after the removal of the larynx.

**Methods:**

We used Internet search engines and libraries to conduct a search of the current medical literature and historical sources of medical information in order to identify and summarize landmark work on this subject.

**Discussion:**

Four types of methods have been used to restore the voices of patients, i.e., external devices, esophageal speech, internal voice prostheses, and surgically created tracheo-esophageal fistulas that do not involve the use of a prosthetic device.

## Introduction

With an estimated number of more than 135,000 new cases occurring annually worldwide, squamous cell carcinoma of the larynx accounts for about 2% of all malignant cancers (1–3). The number of new laryngeal squamous cell carcinoma cases is about 3,000 among men and 400 among women each year in Germany.

Men are affected approximately seven times more often than women. Most cases occur in people aged between 50 and 60 years. Treatment depends on tumor size and may involve transoral laser therapy (TLM), radiotherapy, chemoradiotherapy, and different types of surgical techniques such as the complete or partial removal of the larynx. Partial laryngectomy, i.e., the surgical removal of the affected part of the larynx, is usually an option only for the management of small or medium-sized T1 and T2 tumors. Despite enormous advances in organ preservation surgery, total laryngectomy, i.e., the removal of the entire larynx, is still the treatment of choice for advanced laryngeal and hypopharyngeal carcinoma not amenable for organ preservation therapy.

Total laryngectomy usually involves the removal of all of the thyroid and cricoid cartilages, the arytenoid cartilage, the epiglottis, the hyoid bone, and the prelaryngeal muscles. The pharyngeal tube is closed using a horizontal or T-shaped suture. The cut end of the trachea is sutured to the skin of the neck and an end stoma is thus created.

The removal of the larynx has profound consequences for a patient. The separation of the airway from the mouth, nose, and esophagus leads not only to the loss of the ability to speak but also to the separation of the nasal and pharyngeal segments from the lower airways and thus to the loss of the air conditioning mechanism and active smelling. Patients must learn to cope with a tracheostoma and the associated disadvantages.

Since the loss of the voice organ or, in other words, the loss of normal verbal communication is the most serious consequence of laryngectomy for many patients, total laryngectomy has from early on been associated with attempts to restore the voices of patients. The voice plays an important role in identity. As a result, patients may regard the loss of their voice as a loss of part of their identity.

## The History of Voice Rehabilitation

**Figure 1:**
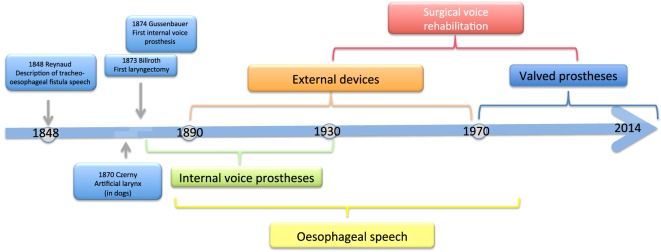
Time bar of voice restoration.

The history of voice rehabilitation begins in the nineteenth century (Figure 1).

As early as 1859, Johann Nepomuk Czermak (1828–1873) developed an artificial larynx and thus laid the foundation for prosthetic voice rehabilitation following total laryngectomy (4). Czermak described the case of an 18-year-old girl with complete laryngeal stenosis who was able to produce a pseudo-whispering voice. In an attempt to increase the loudness of the patient’s voice, Czermak routed the flow of air from the lungs into the lower oropharynx. In 1869, he developed the first artificial larynx (5).

As early as 1870, Vincenz von Czerny (1848–1928), who worked as a surgical assistant in the ENT department of a hospital in Vienna, published his first results with laryngectomies on dogs. From his experience, he concluded that total laryngectomy should also be technically feasible in humans (6). Once he understood that sounds are produced in the larynx but are articulated into speech in the oropharyngeal cavity, he placed the focus of his further research work on the development of an artificial larynx. He developed a cannula that was made by J. Leiter, an instrument maker, and studied its effectiveness in laryngectomized dogs (7).

Methods that enable patients to speak without a larynx were already known in the first half of the nineteenth century. A. A. M. Reynaud, a French military surgeon, described the method of esophageal speech in 1848. He presented a number of cases of traumatic laryngeal stenosis and tracheo-esophageal fistula formation that had been caused by gunshot and fragment injuries (8).

**Figure 2:**
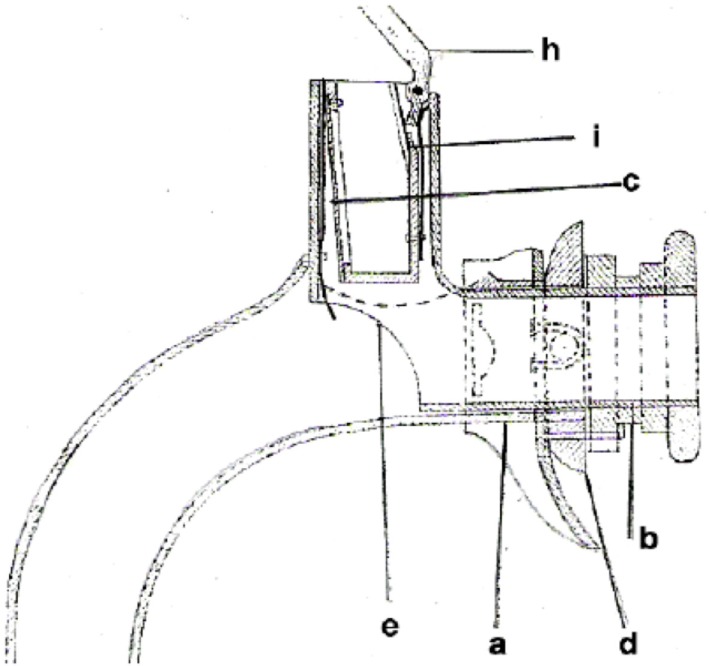
Gussenbauers and Billroths internal artificial larynx. (a) Tracheal cannula, (b) pharyngeal cannula, (c) phonation cannula, (d) turnable sealing, (e) window to trachea, (h) artificial epiglottis, and (i) spring.

Christian Albert Theodor Billroth (1829–1894) is credited with the first successful laryngectomy on a human patient. The procedure was performed on a 36-year-old religious education teacher with an endolaryngeal carcinoma on December 31, 1873. For 3 years, the patient had suffered from hoarseness and aphonia. After the patient had undergone several unsuccessful partial procedures, Billroth decided in November 1873 to perform a vertical partial laryngectomy *via* a thyrotomy in order to remove the tumor. Initially, the patient recovered well, but his condition deteriorated over the next few days. Laryngectomy was then performed. The size of the tumor necessitated the removal of the entire larynx, the epiglottis, and the upper two tracheal rings. The cut end of the trachea was sutured to the skin of the neck, and the pharyngeal opening was narrowed with three sutures. The patient recovered rapidly after surgery. Carl Gussenbauer, one of Billroth’s assistants, was credited with restoring the patient’s voice. At the Third Annual Congress of the German Surgical Society in 1874, Gussenbauer not only described the first documented successful laryngectomy but also presented an internal prosthesis that he had designed for the restoration of voice (9). Having been inspired by the work of Czerny, he developed a phonation cannula together with two instrument makers, namely Leiter (Versions 1 and 2) and Thuerrigl (Version 3). This device was made of hard rubber and consisted of a tracheal cannula, a pharyngeal cannula, and a phonation cannula with a sound-producing metal reed. The tracheal cannula was inserted first, followed by the pharyngeal cannula. This procedure required considerable manual dexterity on the part of the surgeon. The patient was able to produce sounds by digitally occluding the cannula in order to direct air from the trachea to the pharynx. The air caused the metal reed to vibrate and a sound was produced in the pharynx at the level of the base of the tongue. A metal lid was attached to the pharyngeal cannula and served as a pseudo-epiglottis in order to prevent aspiration. The first patient used all three versions of this first voice prosthesis (Figure 2). The first cannula was inserted on the 21st day after surgery.

In 1877, David Foulis, a British surgeon, described an internal voice prosthesis that was largely based on the ideas of Gussenbauer (10). He modified the prosthesis in an attempt to facilitate the insertion of the cannula, improve prosthesis fit, and produce a more natural sounding voice. In contrast to the prosthesis developed by Gussenbauer, the Foulis prosthesis was designed in such a way that first the pharyngeal cannula and then the tracheal cannula were inserted. In addition, the Foulis prosthesis did not have a separate phonation cannula but instead had a metal piece that was introduced into the tracheal cannula and enabled the patient to produce sounds. As a result of this modification, a less metallic voice was created. The metal piece was made of an alloy of silver and copper that produced a richer voice. The length of the pharyngeal cannula was customized to each patient. This approach allowed Foulis to preserve the epiglottis during laryngectomy and thus to ensure the closure of the cranial opening of the pharyngeal cannula during swallowing (11, 12).

**Figure 3:**
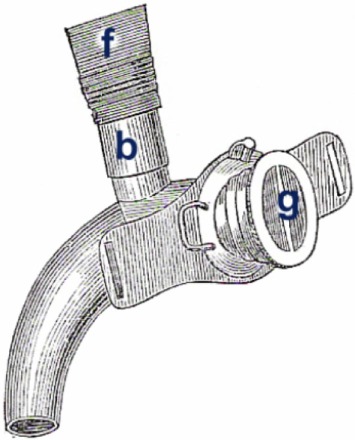
von Bruns artificial larynx. Internal voice prosthesis. (b) rubber tube (f) phonation attachment (g) flap valve.

Victor von Bruns identified several disadvantages of the Gussenbauer prosthesis. The narrow lumina made inspiration and expiration difficult and allowed the patient to wear the prosthesis only for brief periods of time. In addition, the length of the phonation cannula made it almost impossible for patients to eat, chew, or swallow while wearing the prosthesis. Voice failures were common as a result of the accumulation of saliva and mucus in the phonation cannula. Last but not least, the cannula produced a permanent buzzing sound since even quiet breathing caused the metal reed to vibrate. In 1878, Victor von Bruns introduced his version of an internal laryngeal prosthesis that was completely made of new silver (13) (Figure 3). This prosthesis consisted of a tracheal cannula with a wide oval-shaped opening in the curved portion of the cannula, a pharyngeal (or phonation) cannula that was inserted through this opening into the pharynx with perfect fit, and a valve that was attached to the external opening of the tracheal cannula. Unlike the majority of older types of devices that used a simple flap mechanism, this valve consisted of a gutta-percha membrane that was fixed in the middle with two small rods. During inspiration, the sides of the membrane were drawn into the cannula. During expiration, the membrane was pressed against an outer ring that prevented the membrane from being pushed out of the cannula. A membrane-type reed pipe made of rubber was attached to the cranial end of the pharyngeal cannula and thus served as a phonation cannula. The pharyngeal and tracheal cannulas were connected *via* a sliding mechanism, which facilitated the insertion of the prosthesis into the stoma. The major differences between the von Bruns and the Gussenbauer prostheses were the use of a flexible membrane for the production of speech and the use of a phonation cannula that was attached to the cranial end of the pharyngeal cannula and did not constitute a separate element. These modifications facilitated inspiration, and the rubber membrane of the phonation cannula prevented the aspiration of saliva, liquids, and foods from the oropharynx.

Further ideas for improving voice prostheses were published in 1881 by Paul von Bruns, the son of Victor von Bruns (14). In his first attempts, Paul von Bruns modified Richet’s chimney cannula, which consisted of a tracheal cannula with a short pharyngeal tube that was connected to a rubber tube. The phonation tube was closed with a simple cork to prevent the aspiration of liquids and foods during swallowing. This device allowed the patient to produce a pseudo-whispering voice that was strengthened by the flow of air. Von Bruns first modified the handling of the prosthesis. A tracheal cannula was inserted through a pharyngeal element that consisted of a short pharyngeal tube and a shield. A rubber tube that was used for phonation was attached to the pharyngeal tube. As a result of this design, the prosthesis was far easier to handle. Von Bruns further modified the prosthesis by inserting a membrane that had been developed by his father and that allowed patients to produce hands-free speech. He made further modifications to the prosthesis in order to make it easier for patients to assemble the cannula and insert the device into the stoma, for example by using a pharyngeal tube that consisted of several elements and was covered with rubber. It should be noted that von Bruns’ work was a landmark in olfactory and pulmonary rehabilitation. The prosthesis that von Bruns had designed allowed patients to continue to breathe through the mouth and through the nose. Patients were thus able to actively smell and condition inspired air as a result of the preservation of nasopharnyngeal breathing. Von Bruns believed that this air conditioning mechanism played a key role in the prevention of bronchopulmonary infections (15).

In 1892, Julius Wolff developed another internal voice prosthesis. He devised a novel artificial larynx in an attempt to improve the prevention of crusting and improve voice quality. This prosthesis, which was based on the devices described by Paul von Bruns, had a mesh of silver wire that was attached to the end of the pharyngeal cannula (16–18). This mesh allowed air and water to pass through and at the same time protected the device against mucus and food. In addition, Wolff placed the sound-producing flexible rubber reed at a more cranial position in order to improve the resonance of the patient’s voice. A screw allowed the tension of the rubber reed to be adjusted and thus to vary pitch. He increased the opening of the tracheal cannula and modified the valve opening mechanism in order to reduce resistance during inspiration. Wolff reported that this type of prosthesis enabled patients to use appropriate pitch and even to sing.

Eugen Kraus too constructed a voice prosthesis with the primary intention to minimize crusting. Cannulas with vibrating metal reeds were found to be particularly susceptible to this problem since they were affected not only by secretions from the oral and pharyngeal space, as was commonly believed, but also by secretions from the bronchi and the trachea that contaminated the cannula from below. Another problem that was addressed by Kraus was the length of the pharyngeal cannula, which—if it was too short—made phonation difficult as a result of the obliteration of the pharyngeal fistula or—if it was too long—caused oropharyngeal irritation and aspiration. In 1894, Kraus developed a tracheal cannula that was fenestrated in the cranial area. A coil spring that was made of silver and projected upward was soldered to an opening in the cannula and covered with rubber (19). The rubber tube was several millimeters longer than the spring and formed a membrane that vibrated during exhalation. A valve was attached to the tracheal cannula and allowed hands-free speech.

In 1925, R. G. Brown of Australia devised a further internal voice prosthesis. This device included a small metal pitch pipe “G” or “D” that was attached to an ear speculum that was made of gold and had the shape of a cone tapering from 4 to 2.5 mm. The speculum was placed into a tracheo-esophageal fistula and fixed to the neck using a shield. This design enabled the patient to self-insert the prosthesis and remove it before meals (20).

## Pharyngeal Closure

There are case reports in which tracheopharyngeal voice prostheses were used with considerable success. Bottini, for example, reported about a patient who lived with a prosthesis for 38 years. Likewise, Caselli described the case of a female patient who was treated with a voice prosthesis in 1879 and lived with it for a period of 40 years.

These cases, however, were more the exception than the rule since the use of voice prostheses was associated with high complication rates and a morbidity rate of more than 50%. Apart from the constraints of that time, aspiration was the major factor limiting the use of voice prostheses. In those days, the designs of cannulas required a wide pharyngotracheal fistula and were thus associated with a considerable risk of pulmonary aspiration. This problem was successfully addressed by Gluck, Zeller, and Soerensen, who modified the surgical technique in 1881 and the following years and succeeded in completely separating the airway from the digestive tract and in closing the pharyngeal defect. As a result of this new method, mortality was reduced to less than 10%. This approach, however, made the use of tracheal cannulas impossible and required alternative methods of voice rehabilitation (21–23).

## Conservative Methods of Voice Rehabilitation

### Pseudo-Whispering

Pseudo-whispering is a mode of phonation that exclusively uses the air that is present in the oral and pharyngeal space. Appropriate articulation movements allow patients to produce a weak and aphonic voice that enables them to communicate only in a quiet place. As mentioned before, pseudo-whispering was first described by J. N. Czermak (4).

### Esophageal Speech

Esophageal speech is achieved by the intake of air from the oral and pharyngeal space into the upper esophagus, which serves as a reservoir. The air is then released in a controlled manner and causes the pharyngo-esophageal (PE) segment to vibrate for the production of speech.

The use of esophageal speech after laryngectomy was first described by Strübing and Landois in 1889. These authors reported, however, that speech was produced in the region of the base of the tongue (24). In 1896, Störk reported on a large series of patients who were able to use esophageal speech for communication after total laryngectomy (25). He came to the conclusion that voice prostheses were not required for the rehabilitation of speech.

There are different techniques for drawing air into the esophagus.

In 1900, Georg Gottstein was the first to describe a technique in which air is swallowed during inspiration and then regurgitated for speech production. This procedure, however, is ineffective and causes major interruptions of speech (26).

In 1920, Böhme M. Seemann described a technique that involves rapid inhalation. As a result of negative intrathoracic pressure and negative pressure in the esophagus, the upper esophageal sphincter opens and air flows into the esophagus. Once pressure equalization has occurred, the esophageal sphincter closes again and the air that is trapped in the esophagus can be used for phonation (27). Following speech training, patients are sometimes able to create large air reservoirs and speak at a considerable speed.

In 1909, introduced the injection method that was described in detail by Moolenaar-Bijl in the 1950s (28). Patients use their lips, tongue, and buccinator muscle in order to increase intra-oropharyngeal pressure and thus to inject air from the oral space into the esophagus. This air is then used for phonation (29–32).

The segment that produces sounds when esophageal speech is used was identified by Seemann and was described in the work that he published between 1922 and 1926. Seemann defined the PE segment as a pseudoglottis and demonstrated the vibration of this region in radiological examinations. Further important impacts to the investigation of the “pseudoglottis” came from Burger und Kaiser in 1925. They contributed the first scientific evaluation of acoustical parameters of esophageal voice and named this kind of voicing “stomach-ructus-speech.” The investigations included radiographic examination and analysis of phonation time, voicing air volume, pitch, and frequency analysis (33). Investigations that were performed in the 1950s by Damste and Moolenaar-Bijl on esophageal speech and the low complication rates associated with this method were the reason why esophageal speech was the standard of voice rehabilitation after laryngectomy until the mid-1980s. An esophageal voice, however, has two major disadvantages. First, the intake of air into the esophagus leads to frequent interruptions in the flow of speech, and the small esophageal air reservoir allows a patient to accumulate a maximum volume of only 70–100 ml and thus to produce only a few syllables at a time. Second, not all patients master esophageal speech, as was reported by Damste as early as in 1956 (32, 34). Studies performed by Gates in 1982 and by van As and Hilgers in 2004 suggest that only a third of laryngectomized patients are able to learn esophageal speech and use it for satisfactory communication, and only 10% are able to speak clearly (35, 36).

## External Devices

Complete pharyngeal closure required new devices for voice restoration, which can be summarized under the term “external voice prostheses.”

Hochenegg, who succeeded Gussenbauer at the Second Department of Surgery at the Vienna School of Medicine, developed an external speech appliance as early as in 1892. The first version of this prosthesis consisted of a tube that was used to draw air from the tracheostoma through the mouth into the posterior region of the pharynx. The tube was approximately 75 cm long and contained a reed for sound production. The sounds, however, did not result in understandable speech because the air stream was not strong enough and the transoral tube passage made it impossible for the patient to completely close the lips. For this reason, Hochenegg modified the design of the appliance. A bellows, which was located below the axilla, enabled the patient to direct air through the nose into the pharynx using a tube system (37).

Themistokles Gluck, who—together with Zeller and Soerensen—introduced complete pharyngeal closure in order to address the problem of aspiration, constructed a variety of different appliances between 1880 and 1910. In 1910, he described an electromechanical device consisting of a bellows that was powered by one of several electric motors and that was used to direct air either through the nose or through the mouth into the oral space using a tube system with a reed (38). A major disadvantage of this system was the weight of the device and the noise produced by the electric bellows. Another device developed by Gluck was an external pneumatic device powered by pulmonary air. Since this method required a tight seal between the device and the trachea, Gluck attached a piece of rubber to the appliance. The tube system contained a kind of reed with a metal fan that was caused to vibrate by the flow of air and allowed patients to modulate pitch (39, 40).

At the Annual Meeting of the German Society of Surgery in Berlin in 1900, Georg Gottstein presented a speech appliance that consisted of five components. An accessory piece connected the phonation device with the tracheal cannula and a rubber tube connected the accessory piece with a metallic mouth tube. A flap valve with a sound-producing mechanism was placed between the rubber tube and the mouth tube. The sound-producing element contained a metal reed that was caused to vibrate by expiratory air from the trachea. An inflatable air cushion was used to create a tight seal at the tracheostoma. This device enabled patients to produce a relatively loud but monotonous voice, which was the result of the limited frequency range and is a disadvantage of all speech appliances that use a reed for sound production (26).

**Figure 4:**
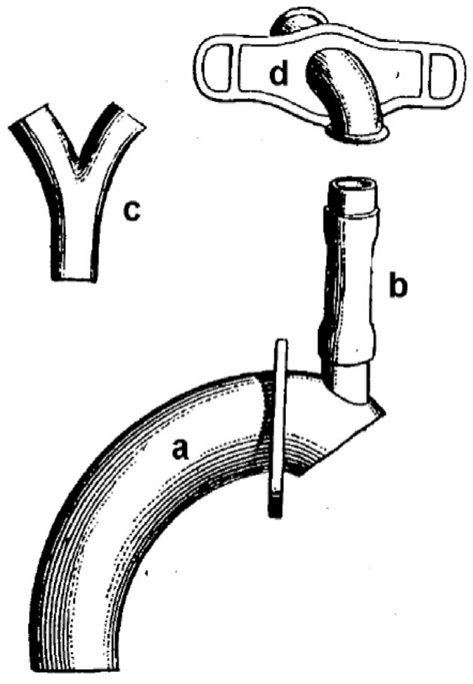
Taptas external voice prosthesis. (a) Tracheal cannula, (b) connecting tube, (c) y-shaped phonation cannula, and (d) neck shield.

In 1900, Nicolas Taptas described an external voice prosthesis that he had used for the first time in an American patient in 1899. This device consisted of an extended cannula with an opening facing upward. A flexible rubber tube was attached to this opening and connected the tracheal portion of the prosthesis with a Y-shaped pharyngeal tube. In order to create a connection between the trachea and the pharynx, Taptas modified the surgical technique. After laryngectomy, he preserved the hyoid and formed a pharyngocutaneous stoma cranial to the tracheostoma. His device thus allowed air to pass from the end tracheal stoma into the pharynx. A flap mechanism was placed between the Y-shaped cannula and the tracheal cannula in order to prevent aspiration (Figure 4) (41).

In 1910, Paul Sudeck devised a voice prosthesis when two of his laryngectomized patients failed to achieve satisfactory voice results using the voice prosthesis developed by Gluck and when he found that the high expiratory pressure required for speech production would eventually cause damage to the lungs. Like Gluck, he developed an external voice prosthesis with a reed. His prosthesis also required the presence of a pharyngostoma that allowed air to pass from the trachea into the oropharynx. When the device was used without the harmonica reed, it allowed patients to produce a pseudo-whispering voice that was strengthened by the larger volume of air. When the device was used with a reed, patients were able to generate a monotonous artificial voice. The pharyngeal tube was removed for eating and sleeping, and the pharyngostoma was closed with a rubber plug (42).

One of the last external voice prostheses that was based on the same principles as those of the above-mentioned devices was developed and described by R. R. Riesz in the United States in 1930. This prosthesis used a reed mechanism and consisted of a cylinder that contained the sound-producing membrane. An external screw allowed the tension of the membrane to be adjusted and thus to vary pitch. Expiratory air was routed through a tracheal cannula into the cylinder where sounds were produced. These sounds were conveyed through a tube into the oral space and used for phonation (43).

In 1936, S. Iglauer reported about an artificial larynx that was a modification of the Riesz prosthesis. This device showed improvements in the regulation of pitch and was easier to handle and clean (44).

In 1965, a method was described by R. T. Barton, which involved the creation of a fistula in the submental region. This fistula connected the superior portion of the trachea and the anterior mouth floor. A T-shaped silicone cannula enabled the patient to direct air into the oral space and produce a pseudo-whispering voice that was strengthened by the flow of air (45).

In 1972, a novel external voice prosthesis was introduced by S. Taub under the name Voice Bak (46, 47). This device was constructed in such a way that patients were able to eat, speak, and breathe without having to make any changes to the prosthesis or without having to remove it. This required a modified surgical technique involving the creation of an end tracheal stoma and an esophagocutaneous fistula at the lower left lateral third of the neck. The fistula tract was fashioned in a retrograde manner and opened into the upper esophagus approximately 2 cm superior to the level of the tracheostoma. The two openings were located at two different levels in order to create a passive flow of air from the tracheostoma to the hypopharynx and at the same time to prevent air from entering the stomach. The main component of the prosthesis was a plastic housing with a flap system that enabled patients to produce hands-free speech. As a result of a physiological increase in pressure during phonation, a bypass valve opened and directed the air flow into the esophagocutaneous fistula, which caused the PE segment to vibrate and enabled patients to produce speech. An adjustable air control valve allowed the device to be adapted to each patient’s individual breathing pressure. The plastic housing was connected to the tracheal cannula *via* an angle piece or fixed with a stoma button in patients who did not tolerate a tracheal cannula. A flexible tube directed the flow of air into the esophagocutaneous fistula. This tube was secured in the fistula using an inflatable cuff. Later versions included a tube that had a cuff with a diameter of approximately 2.5 cm. A flap at the esophageal end of the tube prevented fluid from entering the device.

Also in 1972, D. P. Shedd, an American head and neck surgeon, and his colleagues described another air bypass prosthesis that was intended in particular for patients with extensive pharyngeal resection in whom esophageal speech was not possible (48). The design of the prosthesis was basically similar to that used by Taub. The device consisted of a plastic housing that was located inferior to the tracheostoma and contained a metal piece that vibrated for sound production.

In 1974, N. Edwards introduced a novel technique and external prosthesis on the basis of the experience that Taub and Shedd had gained with the production of voice using an esophagocutaneous fistula (49). Unlike Taub and Shedd, he created an esophagocutaneous fistula that was located superior to the tracheostoma and was directed downward. The fistula tract was formed from pharyngeal mucosa. It was 4–5 cm long and passed obliquely downward from the opening in the skin to the caudal cricopharyngeal region. The external voice prosthesis consisted of two components, i.e., a tracheal cannula whose opening was digitally occluded for phonation and a cannula that was inserted into the esophagocutaneous fistula and that was connected to the tracheal cannula in a circular fashion by two tubes. The tracheal cannula contained a valve mechanism that routed air into the fistula for the production of speech. The cannula that was inserted into the fistula contained a flap that prevented aspiration.

The Tokyo larynx that was modified by Nelson was one of the last external voice prostheses that was placed on the market. The original Tokyo larynx had been developed in the 1960s and consisted of a stoma cover that was connected to two plastic tubes by two swivel joints. A small metal housing with a rubber membrane for sound production was placed between the two tubes. The patient usually had to use both hands in order to operate the device and place a tube in the corner of the mouth for routing air into the oropharynx. The Tokyo larynx was modified by Nelson in 1975. The rubber tubes were replaced by more stable metal tubes and had a further swivel joint. These modifications enabled the patient to operate the device with only one hand (50).

## External Mechanical and Electromechanical Speaking Devices

Patients who are unable to learn esophageal speech or who cannot use a voice prosthesis require other types of speech aids.

The principle underlying external speaking devices is based on an observation that Czermak made in the nineteenth century. He found that patients without a larynx were able to speak when air was directed into the pharynx (pseudo-whispering). One of the first pneumatic speech aids was described by Störk (25). Air was routed from the tracheostoma through the mouth into the pharynx and caused a reed to produce a sound resembling the fundamental frequency of the human voice.

In the twentieth century, these speech aids were replaced by electromechanical appliances. These devices, which are in part based on the early efforts of T. Gluck, included an electrically operated vibrator that caused the muscles and mucosa of the oropharyngeal space to vibrate for sound production (5, 40). There are three main types of electromechanical devices, i.e., external transoral, external transcervical, and intra-oral speech aids. These devices have been used since approximately 1930.

The Cooper-Rand transoral electrolarynx (1957) produced a medium-frequency sound that was routed through a plastic tube into the oropharyngeal space, where it was used for articulation. This speech aid, however, was stigmatizing and was easily affected by saliva. Smoking pipes, such as Ticchioni’s pipe (1959) and the Danapipe, were used to conceal the mechanism and make the device less conspicuous (51).

An external transcervical or neck-type electrolarynx is a device about the size of an electric razor that is held against the neck at the level of the neopharynx or against the floor of the mouth under the mandible. This type of system is easy to handle and does not require extensive maintenance and cleaning. Transcervical electrolarynxes, however, produce monotonous and robot-like speech. Despite this disadvantage, they are still used by patients who are unable to learn esophageal speech and do not tolerate voice prostheses.

The vibrating sound source of an intra-oral speech aid is located inside the oral/pharyngeal space. In 1959, R. V. Tait developed an appliance that was attached to the teeth of a patient (52). A cable that came out of the mouth connected the appliance to an oscillator and a battery. In 1961, H. J. Pichler introduced a system that employed electromagnetic induction and did not require a cable (53). The patient wore one coil around the neck, and a second coil was attached to a tooth. In 1975, R. L. Goode described a device that used radio frequency induction and allowed the patient to vary the frequency of the voice depending on the location and pressure of the transmitter (54). A further electrolarynx that was described by L. D. Lowry in 1981 was a completely self-contained intra-oral device (55).

## Tracheo-Esophageal Fistula

In the 1930s, the idea of creating a tracheo-esophageal fistula for speech production was revisited. In 1932, M. R. Guttman, an American head and neck surgeon, described the case of a laryngectomized patient who had heated an ice pick and created an opening between the trachea and the hypopharynx. This enabled the patient to produce a voice by occluding the tracheostoma with a finger (56). In order to prevent aspiration, the patient used a goose quill to close the fistula when eating and drinking. This case inspired Guttman to develop a puncture technique involving the use of a needle that was connected to a diathermy apparatus. Guttman treated more than 30 patients with this method, which, however, was soon abandoned since it did not provide sufficient protection against aspiration and was associated with a high rate of spontaneous closure of the fistula (57).

Other surgeons too attempted to use surgical approaches to the rehabilitation of speech with tracheo-esophageal shunts. Such methods required a connection between the trachea and the esophagus that was stable enough to prevent spontaneous closure and that at the same time allowed air to be easily drawn into the esophagus and prevented the aspiration of saliva. A further aim was to maintain shunt patency without additional components that required extensive maintenance.

As early as 1942, Briani described a method that involved a second opening that was created superior to the tracheostoma. An epithelialized fistula tract led downward into the esophagus. A valve element that connected the tracheostoma and the fistula allowed the patient to draw air into the esophagus (58, 59).

In 1958, Conley proposed a technique that involved a shunt that was constructed from esophageal mucosa and led downward to the trachea. Air was routed into the esophagus using a special tracheal cannula with a side arm. Obliteration of the fistula tract, however, was a common problem, and a catheter had to be used to preserve the patency of the tract (60).

In 1960, Asai et al. described a complex procedure that involved three stages (61) and was suitable for patients without subglottic tumor extension since it required the preservation of all tracheal rings. The first stage of treatment involved the creation of two tracheostomas. The second stage involved the construction of a pharyngostoma at the level of the base of the tongue. In the third stage, the upper tracheostoma was connected to the pharyngostoma by a tube made of cervical skin. Asai reported that this method led to good phonation results but was associated with a considerable risk of aspiration, which required digital compression of the fistula tract during swallowing. In 1971, the Asai technique was modified by McGrail and Oldfield, who performed a one-stage procedure and used a deltopectoral flap to form a fistula tract (62). A one-stage operation was also described by Calcaterra and Jafek in 1971 and by Komorn in 1974 (63, 64). All techniques, however, had the disadvantage that aspiration was not sufficiently prevented. In 1970, a relatively simple procedure was introduced by Staffieri, which involved the formation of what Staffieri called a phonatory neoglottis (65). Following the creation of a tracheostoma and a tracheal chimney, pharyngeal mucosa was draped and sutured over the end of the cut trachea and a slit was made in the draped portion. Staffieri’s intention was to create an opening that was large enough to allow air to be easily drawn into the pharynx and small enough to prevent aspiration. This, however, was possible in only a few cases.

In 1980, Amatsu described a one-stage technique involving the use of tracheo-esophageal wall mucosa in order to create a fistula between the trachea and the esophagus. This method too failed to reliably prevent aspiration (66, 67).

Subtotal laryngectomy techniques, which, however, were suitable only for patients with tumors without supraglottic and subglottic extension, were described by Arslan and Serafini in 1970, by Mozolewski in 1972, and by Pearson in 1985 (68–70). In each of these techniques, a residual glottis was formed for voicing and a permanent tracheostoma was avoided, if possible. These objectives, however, were achieved only in rare cases since aspiration continued to be the predominant problem.

In the 1990s, surgical methods were revisited, which was mainly attributable to the growing popularity of microvascular surgery. In 1985, Ehrenberger et al. described a technique involving the use of a micro-anastomosed jejunal interposition graft (jejunum siphon) (71). A jejunal tube was attached to the upper tracheal stump and passed upward to the level of the floor of the mouth. Then, the graft was passed downward and sutured end-to-side to the cranial esophagus. Good results were achieved with this technique. Some patients, however, experienced aspiration because of a lowering of the caudal bent portion of the siphon that caused liquid to flow into the other leg of the siphon during swallowing. This technique was modified by Remmert et al. in 1994, who formed a rein from both sides of the neck using the posterior bellies of the biventer muscles. This rein held the bent portion of the siphon and prevented a lowering of the siphon.

Another type of laryngoplasty that involved the use of a revascularized forearm flap and thyroid and auricular cartilage was described for the first time by R. Hagen in 1990 (72, 73). A laryngeal tube was formed from the forearm flap and sutured to the cranial tracheal stump. Using this technique, a wide fistula was created between the trachea and the tongue base and allowed air to be drawn from the trachea into the pharynx. A lid (neo-epiglottis) was formed from the upper end of the laryngeal tube and reinforced with cartilage in order to prevent aspiration. It was positioned at the base of the tongue so that the laryngeal tube was drawn in a cranial and anterior direction and reached a physiological position during swallowing. The authors reported very good voice rehabilitation results in their patients.

In 1994, H. Maier and H. Weidauer described a technique that can be regarded as a modification of the Asai technique (74). They created a long fistula tube that extended from the cranial tracheal stump to the lower tonsillar pole. After laryngectomy, a strip of pharyngeal mucosa with a width of approximately 10 mm was cut from the esophageal opening to the lateral pharynx below the tonsil. The residual pharyngeal mucosa was closed to form a neopharynx using a classic T-shaped suture. A voice fistula was then created using the strip of pharyngeal mucosa and a pedicled pectoralis myofascial flap, which was used to fashion the anterior and lateral walls. Mucosal flap tissue was also used to cover the cranial end of the fistula in order to prevent aspiration. Good results were achieved with this technique. The long fistula, however, necessitated bouginage in some cases. Since this method requires the presence of a sufficient amount of mucosa, it is suitable only for patients with endolaryngeal or very small hypopharyngeal carcinoma.

An innovative approach was reported in 2003 by Kobayashi et al., who used a free ileocecal patch graft. This graft contained the ileocecal valve that was used as a natural valve for the prevention of aspiration (75).

**Figure 5:**
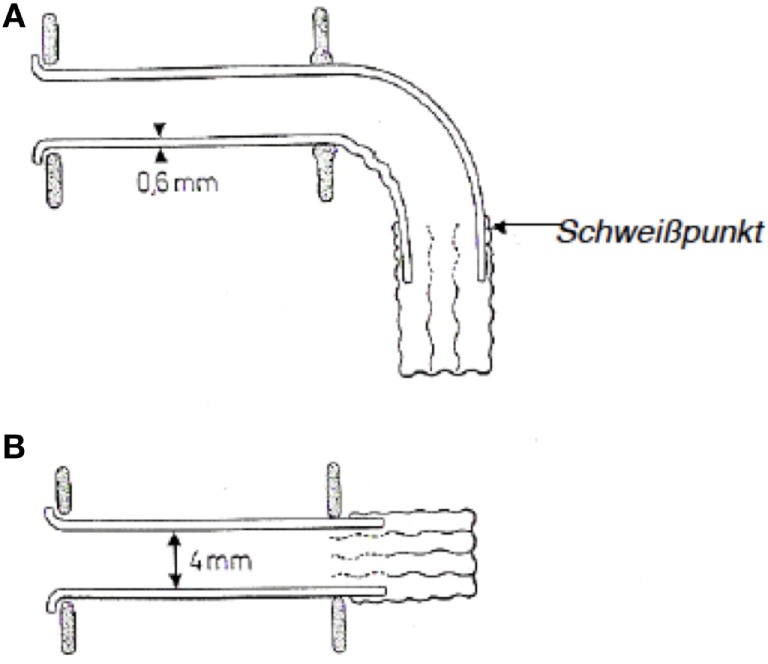
Mozolewski’s first internal voice prosthesis. **(A)** Curved shape and **(B)** straight shape.

In 1972, E. Mozolewski, a Polish otolaryngologist, described the first semi-permanent voice prosthesis that was made from silicone and is considered the first modern voice prosthesis (Figure 5). Mozolewski can therefore be regarded as a pioneer in the field of modern voice rehabilitation after laryngectomy. The voice prosthesis that was developed by Mozolewski and colleagues was manufactured from different materials and customized for every patient. The prosthesis shaft, which had an inner diameter of 3–6 mm and a wall thickness of 0.6 mm, was made from polyethylene or polyvinyl. On the esophageal side of the prosthesis, a valve consisting of two or three layers of a polyethylene foil with a thickness of 0.007 mm was attached to the esophageal flange. The lumen of the polyethylene tube collapsed during swallowing and closed the prosthesis so that aspiration was prevented. A tracheal flange held the prosthesis in place in the tracheo-esophageal fistula. The prosthesis was inserted through the oropharynx using a retrograde technique. Mozolewski’s innovative work and achievements remained unrecognized for a long time because his original paper was written in Polish and a paper that he presented at a congress in Boston in 1981 was not published in the congress proceedings (76–78).

In 1978, Eric D. Blom, an American phoniatrician, and Mark I. Singer, an American head and neck surgeon, began to develop voice prostheses on the basis of the principle introduced by Mozolewski. These prostheses were called duckbill valves (79, 80). In addition, Blom and Singer described a method that involved inserting a voice prosthesis in a secondary procedure by puncturing the posterior tracheal wall. The first Blom–Singer prostheses were non-indwelling prostheses that used a simple flap mechanism and featured a horizontal slit opening in the esophageal end of the prosthesis. In 1981, this type of prosthesis was modified by adding a tracheal flange that prevented a displacement of the prosthesis into the fistula. Since the original design with a slit valve was a frequent cause of prosthesis failure, a Blom–Singer prosthesis with a valve positioned within the tube was introduced commercially in 1983. This type of prosthesis required a lower intrapulmonary pressure and was therefore called a low-pressure prosthesis. Until 1995, all Blom–Singer prostheses were non-indwelling devices. In 1998, modifications to the esophageal and tracheal flanges of Blom–Singer prostheses led to the development of indwelling prostheses that allowed the devices to be inserted in an anterograde fashion. Special prostheses such as the Blom–Singer Advantage prosthesis, which incorporates silver oxide in order to make the device resistant to *Candida* growth, and the Blom–Singer Dual-Valve, which has two valves, were introduced in order to address specific problems such as biofilm formation and valve failure.

Dutch researchers played a leading role in the development of voice prostheses in Europe. In 1980, Nijdam et al. introduced the Groningen voice prostheses, which were the first indwelling prostheses and were placed during either primary or secondary procedures (81, 82). This type of prosthesis was similar to a collar button and consisted of a central tube with a tracheal and an esophageal flange that held the prosthesis in place in the fistula. A crescent-shaped slit on the esophageal side served as a valve mechanism. In 1990, this prosthesis was modified (82). The slit opening was placed at the rim of the tracheal flange and lengthened to 200°. This led to a considerable decrease in phonation pressure. The prosthesis was inserted using a retrograde technique. In the early 1990s, another type of prosthesis was introduced by Nijdam. This prosthesis did not feature a tracheal valve in order to prevent aspiration. Instead, the esophageal end of the fistula was covered by a silicone flange that was large enough to permit the lateral passage of air. Contact between the esophageal flange and the esophageal mucosa prevented aspiration during swallowing.

In 1981, another type of voice prosthesis was introduced by William Panje in the United States. This non-indwelling prosthesis featured differently sized tracheal and esophageal flanges and a valve mechanism consisting of two slits perpendicular to each other (83, 84).

In Germany, a novel type of prosthesis was developed by I. F. Herrmann in 1984. This prosthesis was named after its designer and was known as the ESKA-Herrmann prosthesis (85, 86). It was a non-indwelling device that was based on the Blom–Singer duckbill prosthesis and had a silicone valve. The ESKA-Herrmann prosthesis too had two silicone flanges that held the device in place in the fistula. Unlike other types of prostheses, it had a curved shaft reinforced with a metal tube. A vertical slit opening in the rounded esophageal end of the prosthesis served as a valve. A tracheostoma valve was designed by Herrmann and allowed laryngectomees to produce hands-free speech.

In 1981, a further non-indwelling prosthesis was introduced by Henley-Cohn. A special feature of this device was the large horseshoe-shaped tracheal flange that was used to secure the prosthesis to the skin of the neck (87).

In Italy, an unusual approach was taken by Bonelli in 1982 (88, 89). The Bonelli prosthesis consisted only of a silicone disk and two straps. The disk was inserted through the mouth into the esophagus and secured to the neck using the straps that were passed through the tracheo-esophageal fistula. The pressure of the disk against the esophageal mucosa was altered by changing the tension of the straps. The silicone disk was pressed against the esophageal mucosa during swallowing in order to prevent fluid from passing into the trachea.

A further voice prosthesis was developed by Alberto and Mario Staffieri in Italy in 1986 (90). This prosthesis consisted of a hollow silicone tube with a tracheal flange and a larger esophageal flange. A slit opening in the tracheal end of the prosthesis served as a valve. The prosthesis was inserted in a retrograde fashion. This prosthesis was intended for patients who had undergone a Staffieri procedure and experienced aspiration.

A further voice prosthesis was introduced by Traissac in France in 1986 (91, 92). It consisted of a long esophageal component and a ring-shaped tracheal component. It was inserted through the tracheo-esophageal fistula in a retrograde fashion and secured in position by the tracheal element. Depending on the thickness of the posterior tracheal wall, the shaft of the prosthesis was shortened. The shaft contained a circular flap that served as a valve. This prosthesis, however, was not a success since it was poorly tolerated by patients and difficult to use.

J. Algaba, a Spanish surgeon, developed a further silicone voice prosthesis in 1986 (93). Unlike other types of prostheses, this device had a tracheal flange that was attached to the prosthesis shaft at an angle of 60° in order to improve the fit of the prosthesis in the region of the tracheal mucosa.

In 1988, the first generation of Provox voice prostheses were placed on the market. These indwelling prostheses were developed by Hilgers and Schouwenburg (94) and were similar to a collar button with a tracheal and an esophageal flange. The shaft of the prosthesis contained a silicone disk (flap valve) in order to address the problem of aspiration and was reinforced with an internal radio-opaque hard plastic ring. The prosthesis had a diameter of 22.5 French and was available in different lengths. It was inserted into the fistula tract in a retrograde fashion using a plastic guide wire. This device was novel because it was inserted at the time of laryngectomy (primary prosthetic voice restoration). A further development of this prosthesis was introduced in 1997 and named Provox 2 (95). Apart from a few modifications (reduction in wall thickness), the new prosthesis could be placed either in a retrograde fashion or in an anterograde fashion in awake patients using a special applicator. In 2003, a special prosthesis (ProvoxActivalve) was developed. It was coated with Teflon in order to prevent biofilm growth and was fitted with a magnet to close the valve mechanism (96). In 2010, the Provox prosthesis was further modified (Provox Vega system) in order to reduce air flow resistance and optimize the fit of the prosthesis (97). This device was available with a new tool that facilitated the insertion of the prosthesis (98, 99).

In 1995, a further voice prosthesis was introduced under the name VoiceMaster by Schouwenburg, who attempted to minimize air flow resistance, increase prosthesis lifetime, and optimize ease of use (100). This prosthesis too was made of silicone and had a star-shaped esophageal flange that incorporated a ball valve for the prevention of aspiration.

In Germany, Hagen described the Adeva voice prosthesis in 1998, which was largely based on the design features of available devices. This prosthesis too had two flanges and an internal flap valve. It also featured a small esophageal protective cover (101).

Recent research has focused on the development of a totally implantable voice prosthesis that allows the tracheostoma to be closed in laryngectomized patients. Such a prosthesis was used for the first time by C. Debry in Strasbourg in 2012 (102). The device consisted of two components. During laryngectomy, a titanium tube was placed onto the trachea, which was prepared using a chimney technique. A pectoralis myofascial flap was wrapped around the titanium tube in order to prevent wound healing problems. The tube was positioned in front of the completely closed pharyngeal tube. After healing, the pharyngeal tube was opened at the cranial end of the tube during transoral rigid endoscopy and a flap mechanism was added. Following the closure of the tracheostoma, the patient was able to breathe through the mouth or nose and produce a pseudo-whispering voice. By 2014, three patients had undergone this type of surgery.

## Conclusion

Patients undergoing laryngectomy not only lose their voice and their ability to communicate but also lose an important part of their personality. For this reason, voice rehabilitation has been an integral aspect of treatment after laryngectomy from the very beginning. The focus of prosthetic voice rehabilitation has been on the production of an acceptable voice and the prevention of aspiration, which was a major problem associated with large pharyngostomas. Although the first internal voice prostheses have been the result of brilliant ideas and technically challenging work, they often were of very limited use, especially because of the material they were made of. Hard rubber as well as silver and brass alloys caused skin irritation in many cases, wearing comfort was poor, and many patients had postoperative wound healing problems. The devices were sometimes difficult for patients to use, and saliva and bronchial secretions led to considerable contamination and functional impairment of the prosthesis. Furthermore, the frequency of the voice of patients who used speech appliances with a reed was limited to the frequency of vibration of the sound-producing element.

Once Gluck, Zeller, and Soerensen had introduced complete pharyngeal closure during laryngectomy and thus successfully addressed the problem of aspiration, new external prostheses were required for the vocal rehabilitation of laryngectomized patients. These prostheses directed air from the tracheostoma through a tube system into the oropharynx. The flow of air enabled patients to speak in a whispering voice.

Other prostheses incorporated additional sound-producing elements (reeds), which, however, considerably limited the frequency of the patient’s voice.

As a result of the aforementioned disadvantages, the use of voice prostheses was almost completely discontinued from 1930 to 1970. During that period, the gold standard was esophageal speech, which was associated with a low rate of complications. Only approximately 30% of patients, however, were able to speak in an acceptable manner using this technique.

For this reason, attempts were made to restore voice with surgical techniques, some of which involved complex procedures. In the early 1970s, external voice prostheses enjoyed a brief renaissance. As a result of the size and stigmatizing effect of external prostheses and the difficulties of handling these devices, however, they remained unsuccessful.

**Figure 6:**
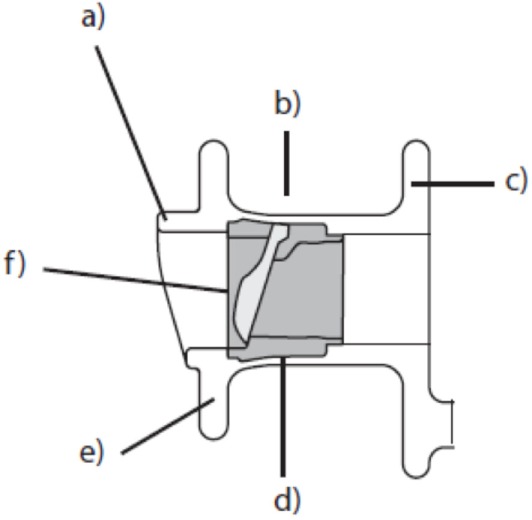
Schematic structure of a modern internal voice prosthesis. (a) Hood, (b) prosthesis shaft, (c) tracheal flange, (d) valve seat, (e) esophageal flange, and (f) valve flap.

The era of modern voice prostheses began in 1972 when Mozolewski described the first internal voice prosthesis that was inserted into a tracheo-esophageal fistula. Since the introduction of new designs by Blom and Singer and Nijdam and Hilgers in the early 1990s, internal voice prostheses have become the gold standard of voice rehabilitation after laryngectomy (Figure 6). Whereas the first prostheses were non-indwelling devices that were removed and re-inserted by the patients themselves, modern prostheses are indwelling and are replaced by medical professionals when required. Constant improvements in the design of voice prostheses led to a decrease in the pressure required for phonation and to better protection against mechanical damage and biofilm growth. Moreover, modern voice rehabilitation systems facilitate comprehensive rehabilitation after laryngectomy since they incorporate elements (heat and moisture exchangers) and automatic tracheostoma valves that allow both vocal and pulmonary rehabilitation to be achieved in laryngectomized patients.

## Author Contributions

KL: literature research, design, and writing of the article.

## Conflict of Interest Statement

The author declares that the research was conducted in the absence of any commercial or financial relationships that could be construed as a potential conflict of interest.
